# Case Report: Inhaled treprostinil: an alternative for the treatment of refractory exacerbation of interstitial lung disease

**DOI:** 10.3389/fmed.2025.1639593

**Published:** 2025-09-05

**Authors:** Francisco León-Román, Beatriz Pintado-Cort, Gabriel Largaespada-Pérez, Hanny G. Khandji-Aslan, Francisco Muñiz-González

**Affiliations:** ^1^Department of Pulmonology, Hospital Recoletas Salud Campo Grande, Valladolid, Spain; ^2^Universidad de Barcelona, Barcelona, Spain; ^3^Department of Cardiology, Hospital Recoletas Salud Campo Grande, Valladolid, Spain; ^4^Intensive Care Unit, Hospital Recoletas Salud Campo Grande, Valladolid, Spain

**Keywords:** treprostinil, exacerbation, interstitial lung disease, treatment, refractory

## Abstract

Acute exacerbation of interstitial lung disease is associated with poor prognosis. Evidence on the best therapeutic strategy for affected patients is lacking. Corticosteroids, broad-spectrum antibiotics, and oxygen therapy are the mainstay of treatment, although immunosuppressants can be added in refractory exacerbations. We present the case of an acute exacerbation in a patient with unclassifiable interstitial lung disease and a history of lymphoma in complete remission. Despite treatment with high-dose corticosteroids, antibiotics, rituximab, and cyclophosphamide, significant hypoxemia persisted, preventing discharge. Echocardiography indicated an intermediate probability of pulmonary hypertension. Subsequent right heart catheterization confirmed precapillary pulmonary hypertension. Inhaled treprostinil was started once other possible causes of pulmonary hypertension had been ruled out. The patient’s condition gradually improved. Treprostinil has been shown to be effective in the treatment of pulmonary hypertension associated with interstitial lung disease. Furthermore, its potential antifibrotic effect is currently being investigated. In the present case, we believe that treating both conditions led to clinical and radiological improvement. However, the limitations of a case report require more robust studies to be performed before firm clinical recommendations can be made.

## 1 Introduction

Acute exacerbation of interstitial lung disease is associated with poor short-term prognosis, and in-hospital mortality can reach 50% in patients with idiopathic pulmonary fibrosis. Acute exacerbations can occur in any subtype of interstitial lung disease, with high morbidity and mortality (1, 2). The diagnostic criteria for acute exacerbation of idiopathic pulmonary fibrosis are used in other interstitial lung diseases and include the following: previous or concurrent diagnosis of interstitial lung disease; acute worsening of respiratory symptoms within less than 1 month; new bilateral ground-glass opacities and/or consolidation superimposed on a background pattern consistent with underlying interstitial lung disease; and exclusion of other causes, such as heart failure and fluid overload (2). Treatment options remain limited, with corticosteroids, broad-spectrum antibiotics, and oxygen therapy as the mainstay, and randomized clinical trials in the field are lacking (2). In cases of refractory exacerbation of interstitial lung disease, immunosuppressive agents could be added to supportive care (3–5). However, no alternatives are available when these treatments prove ineffective. We report the first case in which nebulized treprostinil was a potential alternative in addition to conventional treatment for this condition.

## 2 Case presentation

The patient was a 58 years-old woman with a history of follicular lymphoma treated with R-CHOP in complete remission since 2021 (without requiring radiotherapy). She was being followed up at another center for unclassifiable interstitial lung disease that had been treated with twice-daily oral mycophenolate 500 mg for the previous 4 months. No medical history was available at that time. She had no history of home oxygen therapy.

The patient attended the emergency department with urinary tract infection that did not respond to twice-daily oral ciprofloxacin 250 mg and was admitted for intravenous treatment. No significant changes in her usual respiratory symptoms were reported. Five days later, she developed dry cough, progressive dyspnea, and oxygen saturation below 90%, which improved to 94% with oxygen via nasal cannula at 2 L/min. Auscultation revealed diffuse bilateral crackles, with no extrapulmonary findings, and the vital signs were within normal ranges.

**FIGURE 1:**
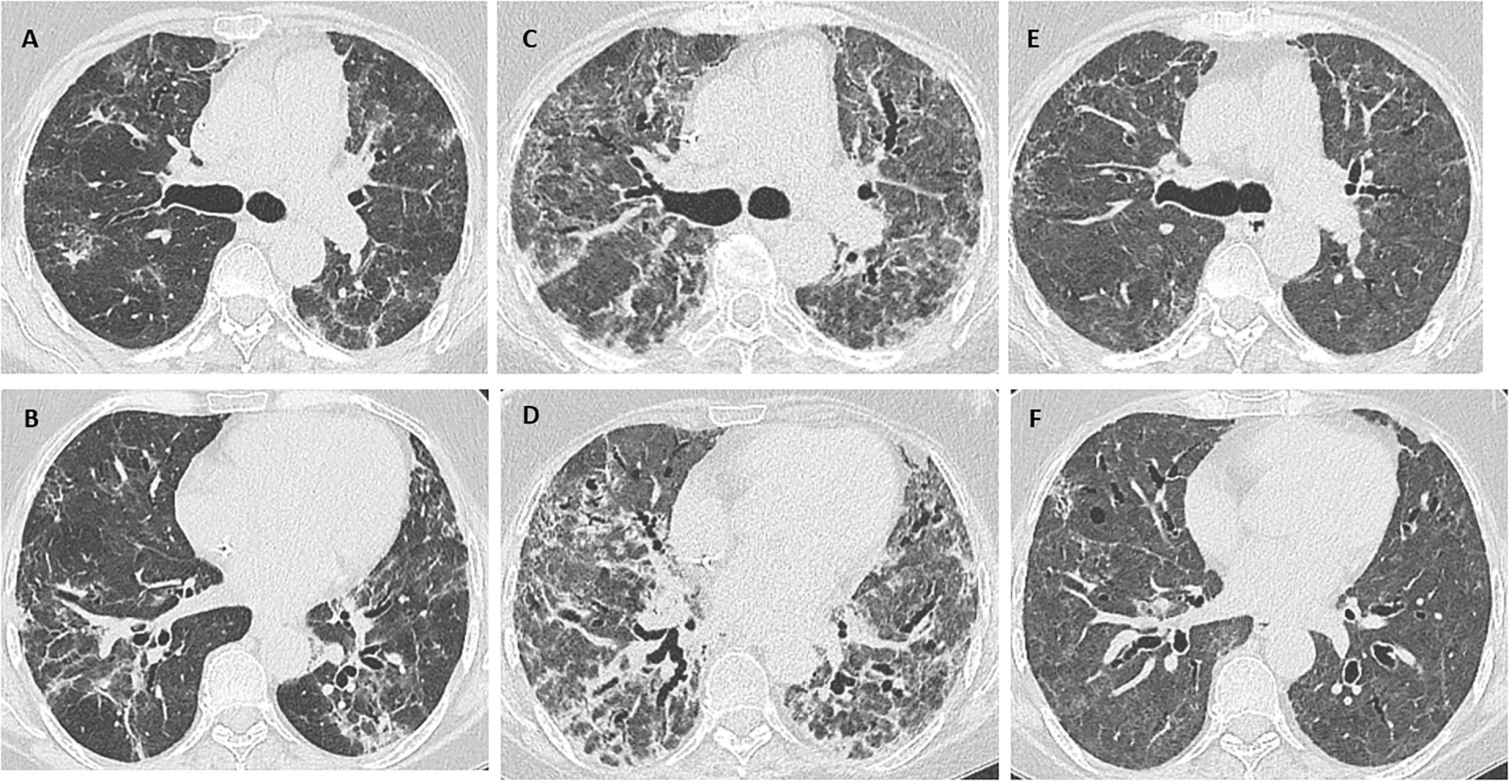
**(A,B)** Chest CT scan: diffuse opacities in both lung fields, possibly associated with organizing pneumonia or an infectious process in the context of underlying fibrotic interstitial lung disease. **(C,D)** Marked worsening of the interstitial lung disease with an associated extensive fibrotic component. **(E,F)** Considerable improvement in interstitial lung disease, with persistent, mildly diffuse ground glass opacity. Note also traction bronchiectasis and bronchiolectasis with no clear areas of honeycombing.

Chest x-ray revealed bilateral diffuse consolidation, and a chest computed tomography scan showed diffuse opacities in both lung fields, possibly associated with organizing pneumonia or an infectious process in the context of underlying fibrotic interstitial lung disease (Figures 1A, B). Given the radiology findings and the suspicion of acute exacerbation of interstitial lung disease, methylprednisolone 1 mg/kg/d, piperacillin-tazobactam, and azithromycin were started, and the patient was referred to the pulmonology department. She reported no workplace or environmental exposure or other regular medication. Serology testing for autoimmunity yielded negative results (Table 1). After a multidisciplinary meeting, we opted for PET-CT to rule out hematologic recurrence.

**TABLE 1:** Relevant laboratory findings.

Blood test results:	WBC 13,500/μL (4,500 to 11,000 WBCs/μL), neutrophils 72%, lymphocytes 18%, eosinophils 1%, monocytes 8%, basophils 1%, Hb 13.9 g/dL (12–16 g/dL), platelets 500,000/μL (150,000–400,000/μL) B-type natriuretic peptide, troponins, procalcitonin, d-dimer, AST, ALT, Cre, Na, K, Cl: normal ranges CRP: 11.8 mg/dL (normal, < 0.9 mg/dL).
Serology:	ANA, ENA, ACE, ANCA, RF, anti-CCP, myositis autoantibodies: negative
Infectious diseases work up:	Gram stain and culture of sputum, pneumococcal and *Legionella* urinary antigen tests, blood cultures, respiratory viral panel, Quantiferon-TB, fungal serology, HCV, HBV, HIV: negative.
Pulmonary function testing after discharge:	FEV_1_, 1.37 L (60%); FVC, 1.53 L (52%); FEV_1_/FVC, 0.89; DLCO, 50%.

WBC, white blood cells; Hb, hemoglobin; CRP, C-reactive protein; ANA, antinuclear antibody; ENA, extractable nuclear antigen antibodies; ACE, angiotensin-converting enzyme; ANCA, antineutrophil cytoplasmic antibodies; RF, rheumatoid factor; Anti-CCP, anti-cyclic citrullinated peptide; HCV, hepatitis C virus; HBV, hepatitis B virus; HIV, human immunodeficiency virus; FEV_1_: forced expiratory volume in 1 s; FVC, forced vital capacity; DLCO, diffusing capacity of the lungs for carbon monoxide.

The scan revealed diffuse uptake in bone, and after consultation with hematology, the patient underwent bone marrow biopsy and aspiration, which excluded recurrence of lymphoma. The patient’s respiratory symptoms worsened 8 days after admission, and she was transferred to the intensive care unit for monitoring and high-flow nasal cannula oxygen therapy (60 L/min and FiO_2_ of 90%), with adequate tolerance and subsequent oxygen saturation of 92%–94%. Bronchoscopy was not performed owing to the patient’s critical condition. The possibility of refractory exacerbation of interstitial lung disease led us to start therapy with rituximab 1 g IV and methylprednisolone 250 mg/d IV for 3 days, followed by gradual tapering.

Her condition progressed slowly, thus requiring continued high-flow nasal cannula oxygen therapy. Endotracheal intubation was not considered appropriate because exacerbation of interstitial lung disease requiring intubation is associated with poor outcomes and high mortality rates. Another dose of rituximab was administered after 14 days, and a new chest CT scan revealed marked worsening of the interstitial lung disease, with an associated extensive fibrotic pattern (Figures 1C, D). As the patient’s condition remained unchanged, cyclophosphamide 500 mg IV was added as a single dose 3 weeks after admission, enabling oxygen therapy to be tapered, and the patient was transferred to the ward.

Despite optimal treatment and respiratory and motor rehabilitation for over 6 weeks, significant hypoxemia persisted. This required supplemental nasal cannula oxygen therapy at 4 L/min, which had to be increased to 60 L/min (FiO_2_ of 100%) with even minimal effort by the patient. Therefore, we performed an echocardiogram, which showed an and intermediate probability of pulmonary hypertension right ventricular dilatation, with no signs suggestive of left heart disease (LVEF, 71%; estimated PASP, 37 mmHg; TR peak velocity, 2.9 m/s; TAPSE, 22 mm; RVEDP, 8 mmHg; no significant alterations for remaining values).

In addition, CT pulmonary angiography ruled out pulmonary embolism, and interstitial lung disease remained unchanged compared to previous radiological studies. Right catheterization was performed (mean pulmonary arterial pressure, 29 mmHg; pulmonary arterial wedge pressure, 11 mmHg; pulmonary vascular resistance, 6.7 Wood units; right atrial pressure, 7 mmHg; cardiac output, 2.7 L/min). After excluding other causes of pulmonary hypertension, we diagnosed the patient with group 3 pulmonary hypertension and started inhaled treprostinil at gradually increasing doses 2 months after admission. Her clinical condition improved, and she tolerated mild exercise.

The patient was discharged with oxygen saturation of 92%–94% requiring nasal cannula oxygen therapy at 2 L/min. One month after discharge, she underwent chest CT (Figures 1E, F) and pulmonary function testing (Table 1). After a multidisciplinary committee discussion, she was eventually diagnosed with fibrosing unclassifiable interstitial lung disease. Biopsy was not considered appropriate because of the patient’s clinical condition, the persistent need for oxygen therapy, and weakness after a 2 months hospital stay. The patient tolerated inhaled treprostinil and mycophenolate 1,000 mg twice daily, and treatment with nintedanib was started. Corticosteroids were slowly tapered to prednisone 5 mg/d. Since recurrence of lymphoma was ruled out and there are no absolute contraindications, the patient is waiting to be evaluated for a lung transplant.

## 3 Discussion

The patient we report had previously been diagnosed with interstitial lung disease and presented respiratory symptoms that worsened during hospitalization. Her chest CT showed ground glass opacity and consolidations. Other relevant pathologies, such as pulmonary embolism, pneumothorax, and heart failure, were excluded (Table 1) (6). Therefore, she was diagnosed with acute exacerbation of interstitial lung disease, which was treated with corticosteroids, oxygen therapy, and broad-spectrum antibiotics according to standard clinical practice. Endotracheal intubation was avoided owing to the poor prognosis associated with intubation in this condition (2, 7).

The poor response to initial treatment led us to add rituximab and, subsequently, cyclophosphamide owing to refractory exacerbation of interstitial lung disease. Both rituximab and cyclophosphamide have been used to stabilize interstitial lung disease and as rescue therapy in acute exacerbations, with a favorable response in case series (3–5). The patient’s clinical condition improved with the aforementioned treatment, although she continued to require high-flow oxygen therapy with minimal exertion. Echocardiographic data suggested an intermediate probability of pulmonary hypertension. Given the extent of fibrosis on the chest CT scan, and after ruling out other causes such as autoimmune diseases, heart disease, and pulmonary embolism, we decided to perform right heart catheterization, which confirmed the diagnosis of group 3 pulmonary hypertension. Since no alternatives were available, treatment was initiated with inhaled treprostinil 1 month after discharge from the ICU. The patient developed mild coughing and occasional headache. No severe adverse effects were observed during follow-up.

Inhaled treprostinil has proven efficacious for treatment of interstitial lung disease–associated pulmonary hypertension, with improved exercise tolerance and increased FVC (8–10). It may also have an antifibrotic effect, which is now being evaluated (11). The antifibrotic effect could be due to the activation of prostaglandin D receptor 1, prostaglandin E receptor 2, and the peroxisome proliferator-activated receptors, which reduce collagen secretion and synthesis and inhibition of fibroblast proliferation (12).

Our case report presents limitations. On the one hand, the patient received multiple treatments, and discriminating between the effect of each on disease progress is difficult. Nevertheless, she did report a significant improvement in symptoms and exercise tolerance days after inhaled treprostinil was initiated. Moreover, a month had passed since the most recent immunosuppressant dose. On the other hand, since treprostinil has proven to be effective in group 3 pulmonary hypertension, it is difficult to differentiate the improvement owing to hemodynamic changes from the possible antifibrotic effect. Finally, as this is a single case report, more studies are needed to assess effectiveness and safety.

Therefore, inhaled treprostinil proved to be a feasible alternative, in addition to standard treatment, for acute exacerbation of interstitial lung disease. Clinical trials are needed to develop an evidence-based treatment for this often fatal condition.

## Data Availability

The original contributions presented in this study are included in this article/supplementary material, further inquiries can be directed to the corresponding author.

## References

[1] Ba C Wang H Jiang C Shi X Jin J Fang Q. Clinical manifestations and prognostic factors analysis of patients hospitalised with acute exacerbation of idiopathic pulmonary fibrosis and other interstitial lung diseases. *BMJ Open Respir Res.* (2024) 11:e001997. 10.1136/bmjresp-2023-001997 38413119 PMC10900369

[2] Collard H Ryerson C Corte T Jenkins G Kondoh Y Lederer D Acute exacerbation of idiopathic pulmonary fibrosis. An international working group report. *Am J Respir Crit Care Med.* (2016) 194:265–75. 10.1164/rccm.201604-0801CI 27299520

[3] Maher T Tudor V Saunders P Gibbons M Fletcher S Denton C Rituximab versus intravenous cyclophosphamide in patients with connective tissue disease-associated interstitial lung disease in the UK (RECITAL): a double-blind, double-dummy, randomised, controlled, phase 2b trial. *Lancet Respir Med.* (2023) 11:45–54. 10.1016/S2213-2600(22)00359-9 36375479

[4] Charokopos A Moua T Ryu J Smischney N. Acute exacerbation of interstitial lung disease in the intensive care unit. *World J Crit Care Med.* (2022) 11:22–32. 10.5492/wjccm.v11.i1.22 35433309 PMC8788209

[5] León Román F Pintado-Cort B García-Casado D Muñiz-González F López García-Asenjo JA Díaz-Rodríguez C Rituximab for the treatment of acute exacerbation of interstitial lung disease associated with connective tissue disease. *RMD Open.* (2023) 9:e003479. 10.1136/rmdopen-2023-003479 37673443 PMC10496654

[6] León-Román F Pintado-Cort B Máiz-Carro L Almonacid-Sánchez C Mercedes-Noboa E Rodríguez-Calle C Acute exacerbation of idiopathic pulmonary fibrosis: an algorithmic approach to diagnosis and management. *J Intern Med.* (2021) 289:930–2. 10.1111/joim.13225 33411961

[7] Kreuter M Polke M Walsh S Krisam J Collard H Chaudhuri N Acute exacerbation of idiopathic pulmonary fibrosis: international survey and call for harmonisation. *Eur Respir J.* (2020) 55:1901760. 10.1183/13993003.01760-2019 32060068

[8] Waxman A Restrepo-Jaramillo R Thenappan T Ravichandran A Engel P Bajwa A Inhaled treprostinil in pulmonary hypertension due to interstitial lung disease. *N Engl J Med.* (2021) 384:325–34. 10.1056/NEJMoa2008470 33440084

[9] Waxman A Restrepo-Jaramillo R Thenappan T Engel P Bajwa A Ravichandran A Long-term inhaled treprostinil for pulmonary hypertension due to interstitial lung disease: increase open-label extension study. *Eur Respir J.* (2023) 61:2202414. 10.1183/13993003.02414-2022 37080567 PMC10307984

[10] Nathan S Deng C King C DuBrock H Elwing J Rajagopal S Inhaled treprostinil dosage in pulmonary hypertension associated with interstitial lung disease and its effects on clinical outcomes. *Chest.* (2023) 163:398–406. 10.1016/j.chest.2022.09.007 36115497 PMC10083130

[11] Nathan S Behr J Cottin V Lancaster L Smith P Deng C Study design and rationale for the TETON phase 3, randomised, controlled clinical trials of inhaled treprostinil in the treatment of idiopathic pulmonary fibrosis. *BMJ Open Respir Res.* (2022) 9:e001310. 10.1136/bmjresp-2022-001310 35787522 PMC9255390

[12] Kolb M Orfanos S Lambers C Flaherty K Masters A Lancaster L The antifibrotic effects of inhaled treprostinil: an emerging option for ILD. *Adv Ther.* (2022) 39:3881–95. 10.1007/s12325-022-02229-8 35781186 PMC9402520

